# Bootstrap-based differential gene expression analysis for RNA-Seq data with and without replicates

**DOI:** 10.1186/1471-2164-15-S8-S2

**Published:** 2014-11-13

**Authors:** Sahar Al Seesi, Yvette Temate Tiagueu, Alexander Zelikovsky, Ion I Măndoiu

**Affiliations:** 1 Computer Science & Engineering Department, University of Connecticut, 06269 Storrs, CT, USA; 2 Computer Science Department, Georgia State University, 34 Peachtree str., 30303 Atlanta, GA, USA

**Keywords:** differential gene expression, bootstrapping, RNA-Seq

## Abstract

A major application of RNA-Seq is to perform differential gene expression analysis. Many tools exist to analyze differentially expressed genes in the presence of biological replicates. Frequently, however, RNA-Seq experiments have no or very few biological replicates and development of methods for detecting differentially expressed genes in these scenarios is still an active research area.

In this paper we introduce a novel method, called IsoDE, for differential gene expression analysis based on bootstrapping. We compared IsoDE against four existing methods (Fisher's exact test, GFOLD, edgeR and Cuffdiff) on RNA-Seq datasets generated using three different sequencing technologies, both with and without replicates. Experiments on MAQC RNA-Seq datasets without replicates show that IsoDE has consistently high accuracy as defined by the qPCR ground truth, frequently higher than that of the compared methods, particularly for low coverage data and at lower fold change thresholds. In experiments on RNA-Seq datasets with up to 7 replicates, IsoDE has also achieved high accuracy. Furthermore, unlike GFOLD and edgeR, IsoDE accuracy varies smoothly with the number of replicates, and is relatively uniform across the entire range of gene expression levels.

The proposed non-parametric method based on bootstrapping has practical running time, and achieves robust performance over a broad range of technologies, number of replicates, sequencing depths, and minimum fold change thresholds.

## Introduction

RNA-Seq has become the new standard for the analysis of differential gene expression [1-3] due to its wider dynamic range and smaller technical variance [4] compared to traditional microarray technologies. However, simply using the raw fold change of the expression levels of a gene across two samples as a measure of differential expression can be unreliable, because it does not account for read mapping uncertainty or capture, fragmentation, and amplification variability in library preparation and sequencing. Therefore, the need for using statistical methods arises. Traditionally, statistical methods rely on the use of replicates to estimate biological and technical variability in the data. Popular methods for analyzing RNA-Seq data with replicates include edgeR [5], DESeq [6], Cuffdiff [7], and the recent NPEBSeq [8].

Unfortunately, due to the still high cost of sequencing, many RNA-Seq studies have no or very few replicates [9]. Methods for performing differential gene expression analysis of RNA-Seq datasets without replicates include variants of Fisher's exact test [4]. Recently, Feng et al. introduced GFOLD [10], a non-parametric empirical Bayesian-based approach, and showed that it outperforms methods designed to work with replicates when used for single replicate datasets.

In this work, we present a novel method for differential gene expression analysis for RNA-Seq data, called IsoDE. Our method uses the traditional bootstrapping approach [11] to resample RNA-Seq reads, in conjunction with the accurate Expectation-Maximization IsoEM algorithm [12] to estimate gene expression levels from the samples. Experimental results on RNA-Seq datasets generated using three different technologies (Illumina, ION Torrent, and 454) from two well-characterized MAQC [13] samples show that IsoDE has consistently high accuracy, comparable or better than that of Fisher's exact test, GFOLD, Cuffdiff, and edgeR (we did not compare directly with NPEBSeq since installation was not successful). Notably, and unlike other methods, IsoDE maintains high accuracy (sensitivity and PPV around 80%) on low coverage RNA-Seq datasets and at lower fold change thresholds.

Recent studies such as Rapaport et al. [14] have reiterated the fact that increasing the number of replicate samples significantly improves detection power over increased sequencing depth. We explored the effect of the number of replicates on prediction accuracy using a RNA-Seq dataset [15] with 7 replicates for each of two conditions (control and E2-treated MCF-7 cells). Although all methods generally benefit from the use of additional replicates, GFOLD and edgeR show a marked discontinuity when transitioning from 1 to 2 replicates. In contrast, IsoDE accuracy varies smoothly with changes in the number of replicates.

## Methods

### Bootstrap sample generation

As most differential expression analysis packages, IsoDE starts with a set *A *of RNA-Seq read alignments for each condition. Bootstrapping can be used in conjunction with any method for estimating individual gene expression levels from aligned RNA-Seq reads, estimation typically expressed in *fragment per kilobase of gene length per million reads *(FPKM). In IsoDE, we use the IsoEM algorithm [16], an expectation-maximization (EM) algorithm that takes into account gene isoforms in the inference process to ensure accurate length normalization. Unlike some of the existing estimation methods, IsoEM uses non-uniquely mapped reads, relying on the distribution of insert sizes and base quality scores (as well as strand and read pairing information if available) to probabilistically infer their origin. Previous experiments have shown that IsoEM yields highly accurate FPKM estimates with lower runtime compared to other commonly used inference algorithms [17].

The first step of IsoDE is to generate *M *bootstrap samples by randomly resampling with replacement from the reads represented in *A*. When a read is selected during resampling, all its alignments from *A *are included in the bootstrap sample. The number of resampled reads in each bootstrap sample equals the total number of reads in the original sample. However, the total number of alignments may differ between bootstrap samples, depending on the number of alignments of selected reads and the number of times each read is selected. The IsoEM algorithm is then run on each bootstrap sample, resulting in *M *FPKM estimates for each gene. The bootstrap sample generation algorithm is summarized below:

1. Sort the alignment file *A *by read ID

2. Compute the number *N *of reads and generate a list  L containing read IDs in the alignment file *A*

3. For *i *= 1*,..., M *do:

(a) Randomly select with replacement *N *read IDs from  L, sort selected read IDs, and extract in *A_i _*all their alignments with one linear pass over *A *(if a read is selected *m *times, its alignments are repeated *m *times in *A_i_*)

(b) Run IsoEM on *A_i _*to get the *i_th _*FPKM estimate for each gene

### Bootstrap-based testing of differential expression

To test for differential expression, IsoDE takes as input two folders which contain FPKM estimates from bootstrap samples generated for the two conditions to be compared. In case of replicates, a list of bootstrap folders can be provided for each condition (one folder per replicate, normally with an equal number of bootstrap samples) - IsoDE will automatically merge the folders for the replicates to get a combined folder per condition, then perform the analysis as in the case without replicates.

In the following we assume that a total of *M *bootstrap samples is generated for each of the compared conditions. We experimented with two approaches for pairing the FPKMs estimated from the two sets of bootstrap samples. In the "matching" approach, a random one-to-one mapping is created between the *M *estimates of first condition and the *M *estimates of the second condition. This results in *M *pairs of FPKM estimates. In the "all" approach, *M*^2 ^pairs of FPKM estimates are generated by pairing each FPKM estimate for first condition with each FPKM estimate for second condition. When pairing FPKM estimate *a_i _*for the first condition with FPKM estimate *b_j _*for the second condition, we use *a_i_*/*b_j _*as an estimate for the fold change in the gene expression level between the two conditions. The "matching" approach thus results in *N *= *M *fold change estimates, while the "all" approach results in *N *= *M*^2 ^fold change estimates.

The IsoDE test for differential expression requires two user specified parameters, namely the minimum fold change *f *and the minimum bootstrap support *b*. For a given threshold *f *(typically selected based on biological considerations), we calculate the percentage of fold change estimates that are equal to or higher than *f *when testing for overexpression, respectively equal to or lower than 1/*f *when testing for underexpression. If this percentage is higher than the minimum bootstrap support *b *specified by the user then the gene is classified as differentially expressed (DE), otherwise the gene is classified as non-differentially expressed (non-DE). The actual bootstrap support for fold change threshold *f *, as well as the minimum fold change with bootstrap support of at least *b *are also included in the IsoDE output to allow the user to easily increase the stringency of the DE test.

**Figure 1:**
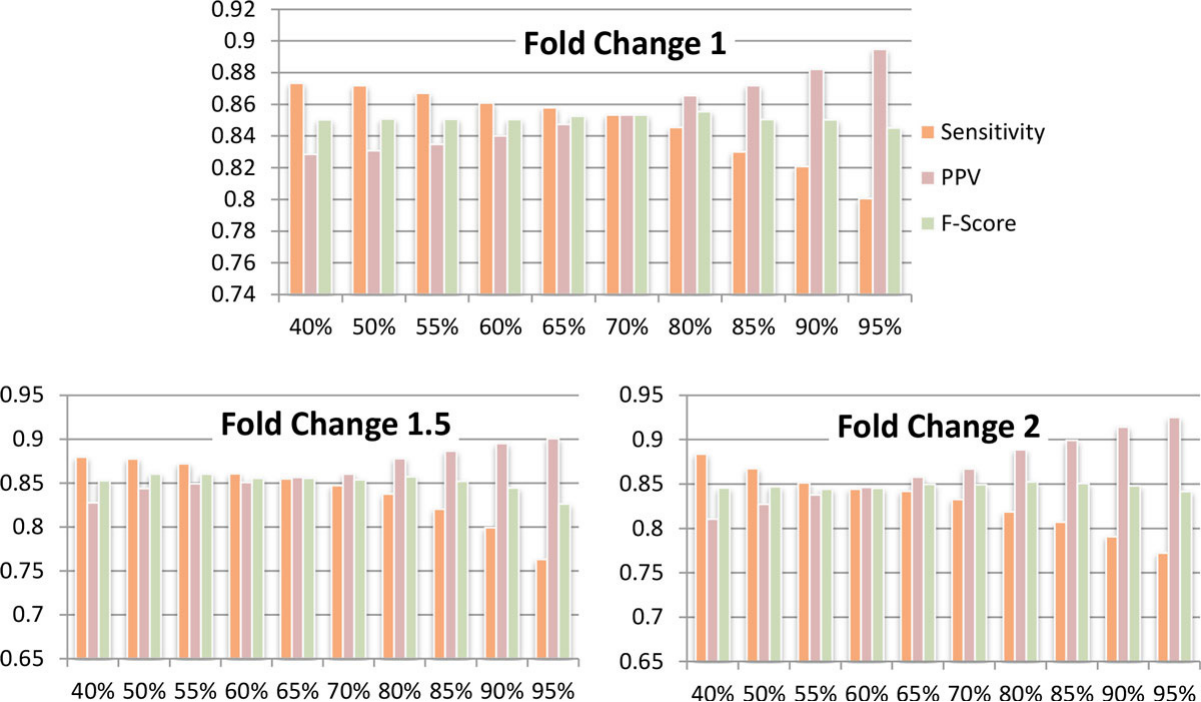
**Sensitivity, PPV, and F-Score of IsoDE-Match (M = 200 bootstrap samples per condition) on the Illumina MAQC data, with varying bootstrap support threshold**.

As discussed in the results section, varying the bootstrap support threshold *b *allows users to achieve a smooth tradeoff between sensitivity and specificity for a fixed fold change *f *(see, e.g., Figure 1). Since different tradeoffs may be desirable in different biological contexts, no threshold *b *is universally applicable. In our experiments we computed *b *using a simple binomial model for the null distribution of fold change estimates and a fixed significance level *α *= 0.05. Specifically, we assume that under the null hypothesis the fold changes obtained from bootstrap estimates are equally likely to be greater or smaller than *f *. We then compute *b *as *x_min_*/*N *, where *x_min _*= min{*x *: *P *(*X *≥ *x*) ≤ *α*} and *X *is a binomial random variable denoting the number of successes in *N *independent Bernoulli trials with success probability of 0.5. For convenience, a calculator for computing the bootstrap support needed to achieve a desired significance level given the (possibly different) numbers of bootstrap samples for each condition has been made available online (see Availability).

The number *M *of bootstrap samples is another parameter that the users of IsoDE must specify. As discussed in the results section, computing the bootstrap support for all genes takes negligible time, and the overall running time of IsoDE is dominated by the time to complete the 2*M *IsoEM runs on bootstrap samples. Hence, the overall runtimes grows linearly with *M *. Experimental results suggest that the "all" pairing approach produces highly accurate results with relatively small values of *M *(e.g., *M *= 20), and thus results in practical runtimes, independent of the number of replicates. We also note that for studies involving pairwise DE analysis of more than two conditions, IsoDE only requires *M *independently generated bootstrap samples per condition. Since the time for computing pairwise bootstrap support values is negligible, the overall running time will grow linearly with the number of conditions.

### Compared methods

The four methods that were compared to IsoDE are briefly described below.

#### Fisher's exact test

Fisher's exact test is a statistical significance test for categorical data which measures the association between two variables. The data is organized in a 2x2 contingency table according to the two variables of interest. We use Fisher's exact test to measure the statistical significance of change in gene expressions between two conditions A and B by setting the two values in the first row of the table to the estimated number of reads mapped per kilobase of gene length (calculated from IsoEM estimated FPKM values) in conditions A and B, respectively. The values in the second row of the contingency table depend on the normalization method used. We compared three normalization methods. The first one is total read normalization, where the total number of mapped reads in conditions A and B are used in the second row. The second is normalization by a housekeeping gene. In this case, the estimated number of reads mapped per kilobase of housekeeping gene length in each condition is used. We also test normalization by ERCCs RNA spike-in controls [18]. FPKMs of ERCCs are aggregated together (similar to aggregating the FPKMs of different transcripts of a gene), and the estimated number of reads mapped per kilobase of ERCC are calculated from the resulting FPKM value and used for normalization. In our experiments, we used POLR2A as a housekeeping gene.

The calculated p-value, which measures the significance of deviation from the null hypothesis that the gene is not differentially expressed, is computed exactly by using the hypergeometric probability of observed or more extreme differences while keeping the marginal sums in the contingency table unchanged. We adjust the resulting p-values for the set of genes being tested using the Benjamini and Hochberg method [19] with 5% false discovery rate (FDR).

#### GFOLD

GFOLD [10] is a generalized fold change algorithm which produces biologically meaningful rankings of differentially expressed genes from RNA-Seq data. GFOLD assigns reliable statistics for expression changes based on the posterior distribution of log fold change. The authors show that GFOLD outperforms other commonly used methods when used for single replicate datasets. We used GFOLD v1.0.7 with default parameters and fold change significance cutoff of 0.05.

#### Cuffdiff

Cuffdiff [7] uses a beta negative binomial distribution model to test the significance of change between samples. The model accounts for both uncertainty resulting from read mapping ambiguity and cross-replicate variability. Cuffdiff reports fold change in gene expression level along with statistical significance. In our comparison, we used Cuffdiff v2.0.1 with default parameters.

#### edgeR

edgeR [5] is a statistical method for differential gene expression analysis which is based on the negative binomial distribution. Although edgeR is primarily designed to work with replicates it can also be run on datasets with no replicates. We used edgeR on counts of uniquely mapped reads, as suggested in [15]. We followed the steps provided in the edgeR manual for RNA-Seq data. calcNormFactors(), estimateCommonDisp(), estimateTagwiseDisp(), and exactTest() were used with default parameter, when processing the MCF-7 replicates. When processing MAQC data and a single replicate of MCF-7 data, estimateTagwiseDisp() was not used, and the dispersion was set to 0 when calling exactTest(). The results where adjusted for multiple testing using the Benjamini and Hochberg method with 5% FDR.

### Mapping RNA-Seq reads

MAQC Illumina reads were mapped onto hg19 Ensembl 63 transcript library; all other datasets were mapped onto hg19 Ensembl 64 transcript library. Illumina datasets (MAQC and MCF-7) were mapped using Bowtie v0.12.8 [20]. ION Torrent reads were mapped using TMAP v2.3.2, and 454 reads were mapped using MOSAIK v 2.1.33 [21]. For edgeR, non-unique alignments were filtered out, and read counts per gene were generated using coverageBed (v2.12.0). Read mapping statistics are detailed in Table S1 in Additional File 1. Number of mapped reads per kilobase of gene length used in Fisher's exact test calculation are based on IsoEM FPKMs.

### Ground truth definition

On MAQC dataset the ground truth was defined based on the available qPCR data from [13]. Each TaqMan assay was run in four replicates for each measured gene. POLR2A (ENSEMBL gene ID ENSG00000181222) was chosen as the reference gene and each replicate CT was subtracted from the average POLR2A CT to give the log2 difference (delta CT). For delta CT calculations, a CT value of 35 was used for any replicate that had CT > 35. The normalized expression value of a gene g would be: 2^{2(CT of POLR2A)-(CT of g)}. We filtered out genes that: (1) were not detected in one or more replicates in each samples or (2) had a standard deviation higher than 25% for the four TaqMan values in each of the two samples. Of the resulting subset, we used in the comparison genes whose TaqMan probe IDs unambiguously mapped to Ensemble gene IDs (686 genes). A gene was considered differentially expressed if the fold change between the average normalized TaqMan expression levels bin the two conditions was greater than a set threshold with the p-value for an unpaired two-tailed T-test (adjusted for 5% FDR) of less than 0.05. We ran the experiment for fold change thresholds of 1, 1.5, and 2.

For experiments with replicates we used the RNA-Seq data generated from E2-treated and control MCF-7 cells in [15]. In this experiment, we compared IsoDE with GFOLD and edgeR. The predictions made by each method when using all 7 replicates for each condition were used as its own ground truth to evaluate predictions made using fewer replicates. The ground truth for IsoDE was generated using a total of 70 bootstrap samples per condition.

### Evaluation metrics

For each evaluated method, genes were classified according to the differential expression confusion matrix detailed in Table 1. Methods were assessed using sensitivity, positive predictive value (PPV), F-score, and accuracy, defined as follows:

**Table 1:** Confusion matrix for differential gene expression

Predicted	Ground truth		
	**Over-Expressed (TOE)**	**Non-Differential (TND)**	**Under-Expressed (TUE)**

Over-Expressed (POE)	TPOE		

Non-Differential (PND)		TPND	

Under-Expressed (PUE)			TPUE

$$Sensitivity=\frac{\left(TPOE+TPUE\right)}{\left(TOE+TUE\right)}$$

$$PPV=\frac{\left(TPOE+TPUE\right)}{\left(POE+PUE\right)}$$

$$Accuracy=\frac{\left(TPOE+TPND+TPUE\right)}{\left(TOE+TND+TUE\right)}$$

$$F-score=2\times \frac{Sensitivity\times PPV}{Sensitivity+PPV}$$

## Results and discussion

### Datasets

We conducted experiments on publicly available RNA-Seq datasets generated from two commercially available reference RNA samples and a breast cancer cell line.

To compare the accuracy of different methods, we used RNA-Seq data RNA samples that were well-characterized by quantitative real time PCR (qRT-PCR) as part of the MicroArray Quality Control Consortium (MAQC) [13]; namely an Ambion Human Brain Reference RNA, Catalog # 6050), henceforth referred to as HBRR and a Stratagene Universal Human Reference RNA (Catalog # 740000) henceforth referred to as UHRR. To assess accuracy, DE calls obtained from RNA-Seq data were compared against those obtained as described in the Methods section from TaqMan qRT-PCR measurements collected as part of the MAQC project (GEO accession GPL4097).

We used RNA-Seq data generated for HBRR and UHRR using three different technologies: Illumina, ION-Torrent, and 454. Details about the datasets and their SRA accession numbers (or run IDs for ION Torrent datasets) are available in Table S1 in Additional File 1.

The MCF-7 RNA-Seq data was generated (from the MCF-7 ATCC human breast cancer cell line) by Liu et al. [15] using Illumina single-end sequencing with read length of 50 bp. A total of 14 biological replicates were sequenced from two conditions: 7 replicates for the control group and 7 replicates for E2-treated MCF-7 cells. Sequencing each replicate resulted produced between 25 and 65 millions of mapped reads. Details about this dataset and accession numbers are also available in Table S1 in Additional File 1.

### Bootstrapping support and pairing strategy effects on IsoDE accuracy and runtime

We evaluated both the "matching" and "all" pairing strategies of IsoDE (referred to as IsoDE-Match and IsoDE-All) for fold change threshold *f *of 1, 1.5, respectively 2, and bootstrap support threshold *b *between 40% and 95%. The results of IsoDE-Match with *M *= 200 bootstrap replicates per condition are shown in Figure 1. The results show that, for each tested value of *f *, varying *b *results in a smooth tradeoff between sensitivity and PPV, while the F-score changes very little. For the remaining experiments we used a bootstrap support level *b *computed using a significance level of 0.05 under the binomial null model detailed in the Methods section. Note that the value of *b *selected in this way depends on the number *N *of fold change estimates, which in turn depends on both *M *and the pairing strategy (*N *is equal to *M *for IsoDE-Match, respectively to *M*^2 ^for IsoDE-All).

To determine the best pairing strategy, we ran IsoDE-Match and IsoDE-All with number of bootstrap samples *M *varying between 10 and 200 (results not shown). For the considered measures, IsoDE-All achieved an accuracy very close to that of IsoDE-Match when run with a comparable value of *N *. For example, as shown in Tables 2, 3, 4, IsoDE-All run on *M *= 20 bootstrap samples (*N *= 400) had similar accuracy with the largest number of bootstrap samples we could use with IsoDE-Match (*M *= *N *= 200).

**Table 2:** Accuracy, sensitivity, PPV and F-Score in % for MAQC Illumina dataset and fold change threshold *f *of 1, 1.5, and 2.

Fold Change	Method	Accuracy %	Sensitivity %	PPV %	F-Score %
	FishersTotal	70.41%	70.79%	91.24%	79.72%
	FishersHousekeeping	65.60%	65.22%	95.05%	77.36%
1	GFOLD	78.13%	80.06%	92.67%	**85.90%**
	Cuffdiff	11.37%	6.96%	**100.00%**	13.01%
	edgeR	73.03%	73.26%	95.56%	82.94%
	IsoDE-Match	**82.63%**	**87.46%**	83.70%	85.54%
	IsoDE-All	82.22%	87.17%	82.82%	84.94%

	FishersTotal	74.05%	78.20%	84.85%	81.39%
	FishersHousekeeping	76.68%	73.61%	93.67%	82.44%
1.5	GFOLD	79.15%	79.35%	90.41%	84.52%
	Cuffdiff	28.43%	8.60%	**100.00%**	15.85%
	edgeR	**80.01%**	79.92%	92.07%	**85.57%**
	IsoDE-Match	78.98%	86.23%	84.62%	85.42%
	IsoDE-All	79.01%	**86.42%**	84.49%	85.44%

	FishersTotal	78.43%	81.86%	82.44%	82.15%
	FishersHousekeeping	81.20%	80.00%	88.21%	83.90%
2	GFOLD	82.94%	78.84%	92.37%	85.07%
	Cuffdiff	40.96%	10.47%	**100.00%**	18.95%
	edgeR	**83.67%**	81.63%	91.17%	**86.13%**
	IsoDE-Match	82.04%	85.58%	85.19%	85.38%
	IsoDE-All	81.20%	**86.74%**	83.07%	84.87%

The number of bootstrap samples is *M *= 200 for IsoDE-Match and *M *= 20 for IsoDE-All, and bootstrap support was determined using the binomial model with significance level *α *= 0.05.

**Table 3:** Accuracy, sensitivity, PPV and F-Score in % for Ion Torrent dataset and fold change threshold *f *of 1, 1.5, and 2.

Fold Change	Method	Accuracy %	Sensitivity %	PPV %	F-Score %
	FisherTotal	71.68%	72.76%	90.56%	80.69%
	FisherHousekeeping	67.15%	66.87%	**94.74%**	78.40%
	FisherERCC	71.39%	72.45%	88.97%	79.86%
1	GFOLD	75.77%	77.55%	90.43%	83.50%
	IsoDE-Match	**81.75%**	**86.38%**	82.18%	**84.05%**
	IsoDE-All	81.46%	86.07%	82.13%	**84.05%**

	FisherTotal	74.16%	78.39%	85.06%	81.59%
	FisherHousekeeping	76.06%	73.23%	**92.96%**	81.93%
	FisherERCC	74.31%	78.59%	85.45%	81.87%
1.5	GFOLD	75.47%	77.63%	87.88%	82.44%
	IsoDE-Match	77.66%	83.94%	84.75%	**84.34%**
	IsoDE-All	**77.81%**	**84.13%**	84.45%	84.29%

	FisherTotal	79.71%	83.02%	84.00%	83.51%
	FisherHousekeeping	**81.75%**	80.70%	88.75%	84.53%
	FisherERCC	79.42%	82.56%	84.12%	83.33%
2	GFOLD	80.58%	76.74%	**90.66%**	83.12%
	IsoDE-Match	**81.75%**	85.81%	84.63%	**85.22%**
	IsoDE-All	81.61%	**86.28%**	84.13%	85.19%

The number of bootstrap samples is *M *= 200 for IsoDE-Match and *M *= 20 for IsoDE-All, and bootstrap support was determined using the binomial model with significance level *α *= 0.05.

**Table 4:** Accuracy, sensitivity, PPV and F-Score in % for the First 454 dataset and fold change threshold *f *of 1, 1.5, and 2.

Fold Change	Method	Accuracy %	Sensitivity %	PPV %	F-Score %
	FisherTotal	34.01%	30.50%	95.63%	46.24%
	FisherHousekeeping	24.52%	20.12%	**94.74%**	33.38%
1	GFOLD	55.62%	54.18%	92.11%	68.23%
	IsoDE-Match	75.33%	79.57%	77.41%	78.47%
	IsoDE-All	**78.85%**	**84.67%**	81.04%	**82.82%**

	FisherTotal	48.18%	35.37%	89.81%	50.75%
	FisherHousekeeping	42.48%	24.86%	**97.74%**	39.63%
1.5	GFOLD	62.19%	58.13%	85.39%	69.17%
	IsoDE-Match	64.09%	74.19%	72.52%	73.35%
	IsoDE-All	**72.85%**	**79.54%**	80.62%	**80.08%**

	FisherTotal	57.96%	39.53%	85.43%	54.05%
	FisherHousekeeping	55.33%	29.30%	**97.67%**	45.08%
2	GFOLD	69.05%	61.16%	83.49%	70.60%
	IsoDE-Match	67.15%	76.51%	70.30%	73.27%
	IsoDE-All	**75.18%**	**80.93%**	78.03%	**79.45%**

The number of bootstrap samples is *M *= 200 for IsoDE-Match and *M *= 20 for IsoDE-All, and bootstrap support was determined using the binomial model with significance level *α *= 0.05.

**Figure 2:**
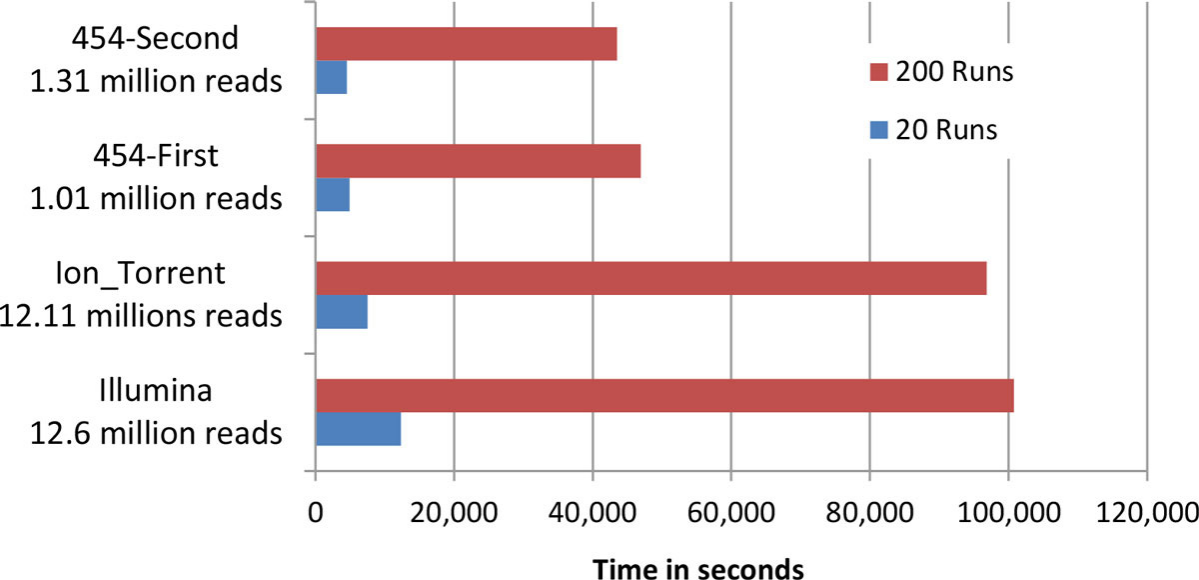
**Running times (in seconds) of IsoDE-Match with *M *= 200 and IsoDE-All with *M *= 20 on several MAQC datasets**. The indicated number of reads represents the total number of mapped reads over both conditions of each dataset, for more information on the datasets see Table S1.

Since for a fixed *N *IsoDE-Match requires 2*N *bootstrap samples while IsoDE-All requires only $2\sqrt{N}$ of them, using IsoDE-All is significantly faster in practice. Indeed, most of the IsoDE time is spent generating bootstrap samples and estimating expression levels for each of them using the IsoEM algorithm, with bootstrap support computation typically taking a fraction of a minute. Figure 2 shows the time required to generate *M *= 20, respectively *M *= 200, bootstrap samples for both conditions of several MAQC datasets. All timing experiments were conducted on a Dell PowerEdge R815 server with quad 2.5 GHz 16-core AMD Opteron 6380 processors and 256 Gb RAM running under Ubuntu 12.04 LTS. IsoEM is run on bootstrap samples sequentially, but for each run its multi-threaded code takes advantage of all available cores (up to 64 in our experimental setup). As expected, the running time scales linearly with the number of bootstrap samples per condition, and thus generating *M *= 20 bootstrap samples per condition is nearly 10 times faster than generating *M *= 200 of them. Overall, IsoDE-Match with *M *= 20 has reasonable running time, varying between 1 hour for the smallest 454 dataset to 3.5 hours for the Illumina dataset.

### Results for DE prediction without replicates

We compared IsoDE against GFOLD, Cuffdiff, edgeR, and different normalization methods for Fisher's exact test; namely total normalization, housekeeping gene (POLR2A) normalization, and normalization using External RNA Controls Consortium (ERCC) RNA spike-in controls [18]. Cuffdiff results were considerably worse on the Illumina MAQC dataset, compared to other methods. Consequently, Cuffdiff was not included in other comparisons. edgeR was also not included in further comparisons due to lack of clear definition of uniquely mapped reads for ION-Torrent and 454 datasets which were mapped using tools based on local alignment algorithms. ERCC spike-ins were available only for ION Torrent samples; therefore, ERCC normalization for Fisher's exact test was conducted only for ION Torrent datasets.

Table 2 shows the results obtained for the MAQC Illumina dataset using minimum fold change threshold *f *of 1, 1.5, and 2, respectively. Table 3 shows the results obtained from combining the ION Torrent runs listed in Table S1 (Additional File 1) for each of the MAQC datasets using the same values of *f *. Table 4 shows the results for the First 454 MAQC dataset, while results for the Second 454 dataset are presented in Table S2 in Additional File 1. For each fold change threshold, the best performing method for each statistic is highlighted in bold.

IsoDE has very robust performance, comparable or better than that of the other methods for differential gene expression. Indeed, IsoDE outperforms them in a large number of cases, across datasets and fold change thresholds. Very importantly, unlike GFOLD and Fisher's exact test, IsoDE maintains high accuracy (sensitivity and PPV around 80%) on datasets with small numbers of mapped reads such as the two 454 datasets. This observation is confirmed on results obtained for pairs of individual ION-Torrent runs, presented in Tables S3 and S4 in Additional File 1. This makes IsoDE particularly attractive for such low coverage RNA-Seq datasets.

### DE prediction with replicates

**Figure 3:**
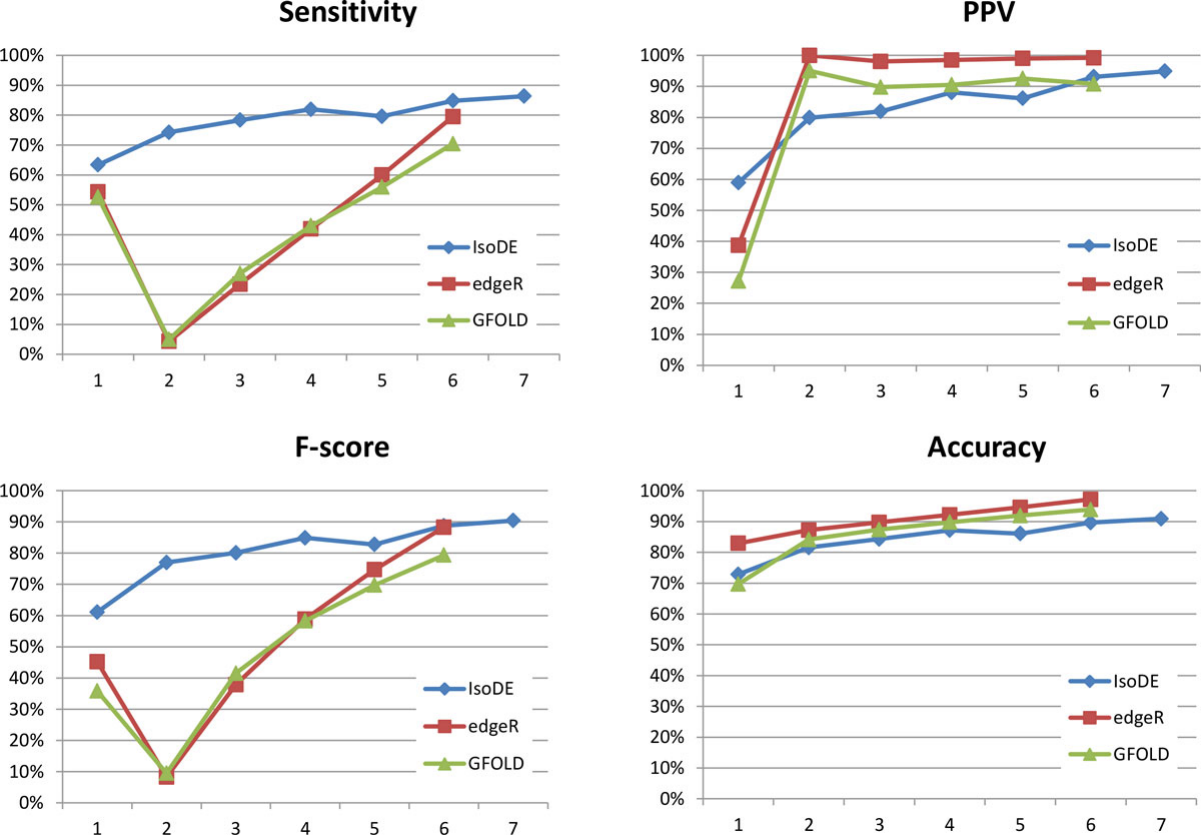
**Sensitivity, PPV, F-Score, and accuracy of IsoDE-All (with 20 bootstrap runs per condition), edgeR, and GFOLD on the Illumina MCF-7 data with minimum fold change of 1 and varying number of replicates**.

We also studied the effect of the number of biological replicates on prediction accuracy using the MCF-7 dataset. We performed DE predictions using an increasing number of replicates. IsoDE was run with a total of 20 bootstrap samples per condition, distributed equally (or as close to equally as possible) among the replicates, as detailed in Table S5. GFOLD and edgeR were evaluated for 1 through 6 replicates using as ground truth the results obtained by running each method on all 7 replicates (see the Methods section). For IsoDE, we also include the results using *M *= 20 bootstrap samples from all 7 replicates as its ground truth is generated using a much larger number of bootstrap samples (*M *= 70). Figure 3 shows the results of the three compared methods for a fold change threshold of 1, results for fold change thresholds 1.5 and 2 are shown in Figures S1 and S2 in Additional File 1.

Since for this experiment the ground truth was defined independently for each method, it is not meaningful to directly compare accuracy metrics of different methods. Instead, we focus on the rate of change in the accuracy of each method as additional replicates are added. Generally, all methods perform better when increasing the number of replicates. However, the accuracy of IsoDE varies smoothly, and is much less sensitive to small changes in the number of replicates. Surprisingly, this is not the case for GFOLD and edgeR sensitivity, which drops considerably when transitioning from 1 to 2 replicates, most likely due to the different statistical models employed with and without replicates. Although we varied the number of replicates without controlling the total number of reads as Liu et al. [15], our results strongly suggest that cost effectiveness metrics such as those proposed in [15] are likely to depend on to the specific method used for performing DE analysis. Therefore, the analysis method should be taken into account when using such a metric to guide the design of RNA-Seq experiments.

### Effect of gene abundance

We also studied the effect of gene abundance on the IsoDE, GFOLD, and edgeR prediction accuracy. We selected the subset of genes that are expressed in at least one of the two RNA samples. We sorted these genes by the average of the gene's expression. We used the FPKM values predicted by IsoEM, the FPKM values predicted by GFOLD, and the number of uniquely mapped reads, for IsoDE, GFOLD, and edgeR, respectively. The genes were then divided into quintiles, for each method independently, where quintile 1 had the genes with the lowest expression levels, and quintile 5 had the genes with the highest expression levels. Sensitivity, PPV, and F-score where calculated for each quintile separately.

**Figure 4:**
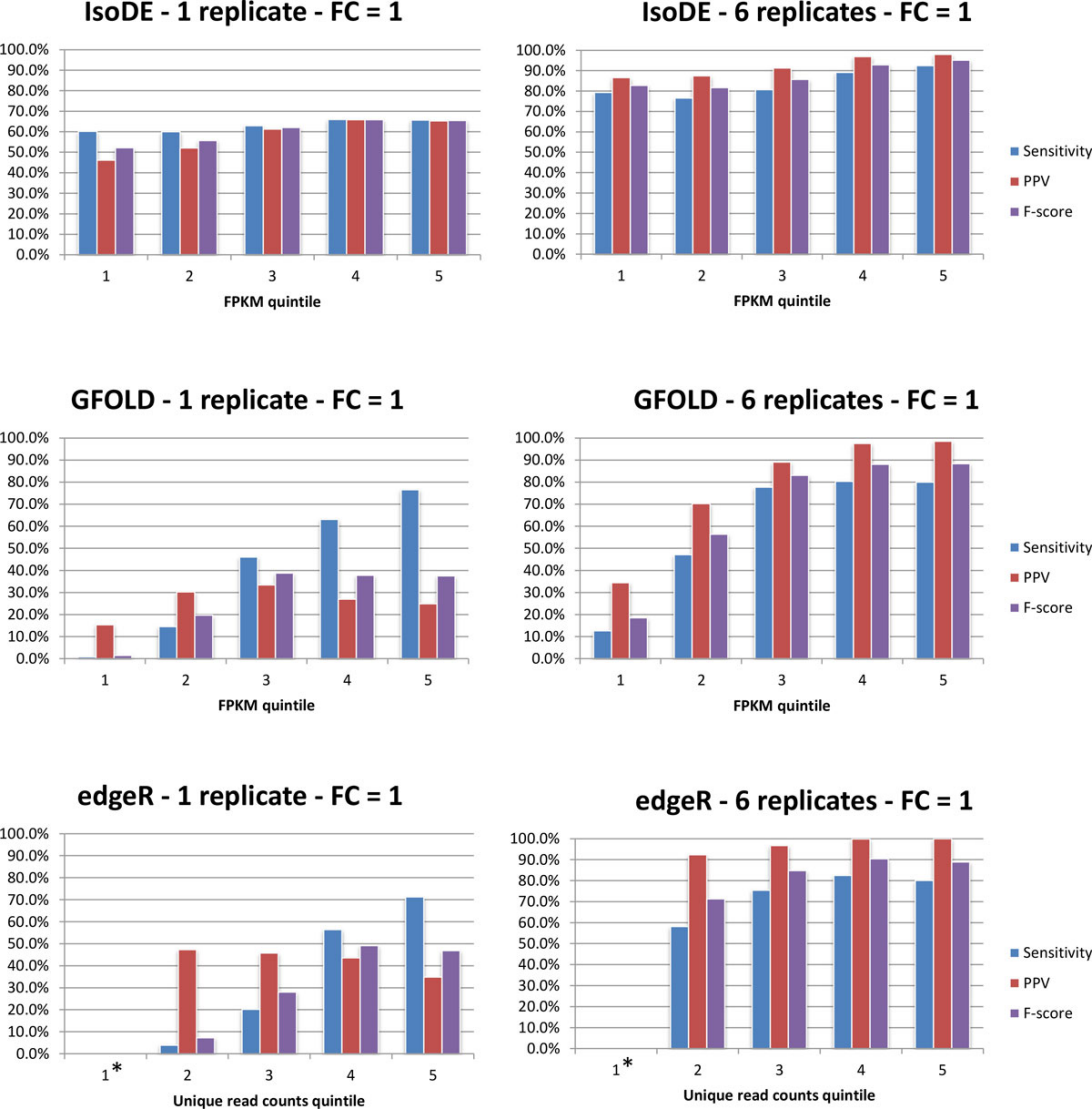
**Sensitivity, PPV, and F-Score of IsoDE-All (with 20 bootstrap runs per condition), edgeR, and GFOLD on the Illumina MCF-7 data, computed for quintiles of expressed genes after sorting in non-decreasing order of average FPKM for IsoDE and GFOLD and average count of uniquely aligned reads for edgeR**. First quintile of edgeR had 0 differentially expressed genes according to the ground truth (obtained by using all 7 replicates).

Figure 4 shows that, for results with both 1 and 6 replicates, sensitivity, PPV, and F-score of IsoDE are only slightly lower on genes with low expression levels compared to highly expressed genes (similar results are achieved for intermediate numbers of replicates and higher fold change thresholds). In contrast, GFOLD shows a marked difference in all accuracy measures for genes in the lower quintiles compared to those in the higher quintiles. The sensitivity of edgeR is also lower for genes expressed at low levels, however it's PPV is relatively constant across expression levels.

## Conclusions

A practical bootstrapping based method, IsoDE, was developed for analysis of differentially expressed genes in RNA-Seq datasets. Unlike other existing methods, IsoDE is non-parametric, i.e., does not assume an underlying statistical distribution of the data. Experimental results on publicly available datasets both with and without replicates show that IsoDE has robust performance over a wide range of technologies, sequencing depths, and minimum fold changes. IsoDE performs particularly well on low coverage RNA-Seq datasets, at low fold change thresholds, and when no or very few replicates are available.

## Availability

IsoDE has been implemented in Java and can be run on any platform with a Java virtual machine. The source code and installation instructions are available at http://dna.engr.uconn.edu/software/IsoDE/. A web-based calculator for computing the bootstrap support based on the desired number of bootstrap samples and significance level is available at http://dna.engr.uconn.edu/~software/cgi-bin/calc/calc.cgi.

## Competing interests

The authors declare that they have no competing interests.

## Authors' contributions

SS implemented and tested Fisher's exact test, prepared the testing data used to generate the experimental results, ran Cuffdiff, GFOLD, and edgeR, and performed comparisons between these methods and IsoDE. SS also contributed to designing the algorithms and wrote part of the manuscript. YTT developed, implemented and tested the algorithms for IsoDE and wrote part of the manuscript. AZ contributed to designing the algorithms and the experiments, writing the manuscript and supervised the project. IM contributed to designing the algorithms and the experiments, writing the manuscript and supervised the project. All authors read and approved the final manuscript.

## Supplementary Material

Additional file 1 **Supplementary figures and tables are supplied in PDF format**. Click here for file

## Declarations

Publication costs for this work were funded by the corresponding authors' institution.

This article has been published as part of *BMC Genomics *Volume 15 Supplement 8, 2014: Selected articles from the 9th International Symposium on Bioinformatics Research and Applications (ISBRA'13): Genomics. The full contents of the supplement are available online at http://www.biomedcentral.com/bmcgenomics/supplements/15/S8.
