# Supplementary material for: Bootstrap-based differential gene expression analysis for RNA-Seq data with and without replicates
Source: BMC Genomics. 2014 Nov 13;15(Suppl 8):S2. doi: 10.1186/1471-2164-15-S8-S2 (PMC4248812; doi:10.1186/1471-2164-15-S8-S2)
Supplement: Additional file 1 — Supplementary figures and tables are supplied in PDF format. [file 1471-2164-15-S8-S2-S1.pdf]

Supplementary figures and tables

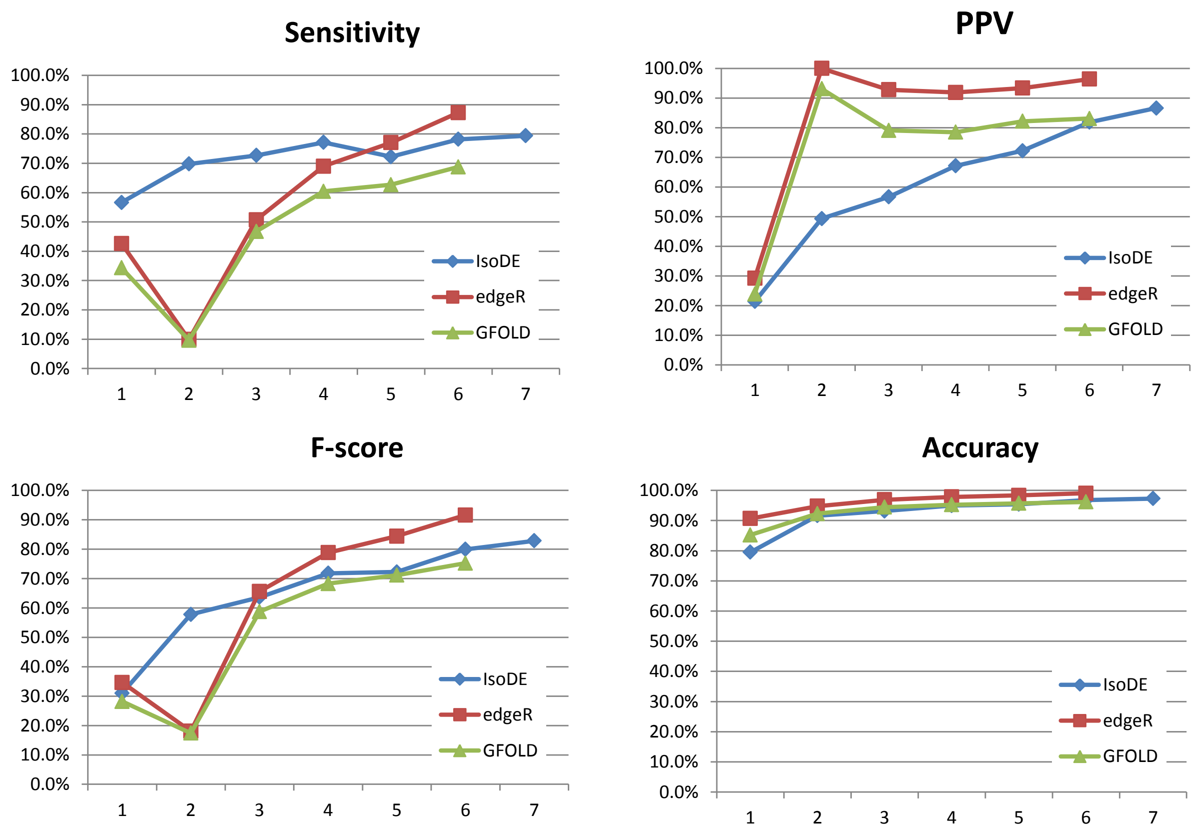

Figure S1: Sensitivity, PPV, F-Score, and accuracy of IsoDE-All (with 20 bootstrap runs per condition), edgeR, and GFOLD on the Illumina MCF-7 data with varying number of replicates and minimum fold change 1.5.

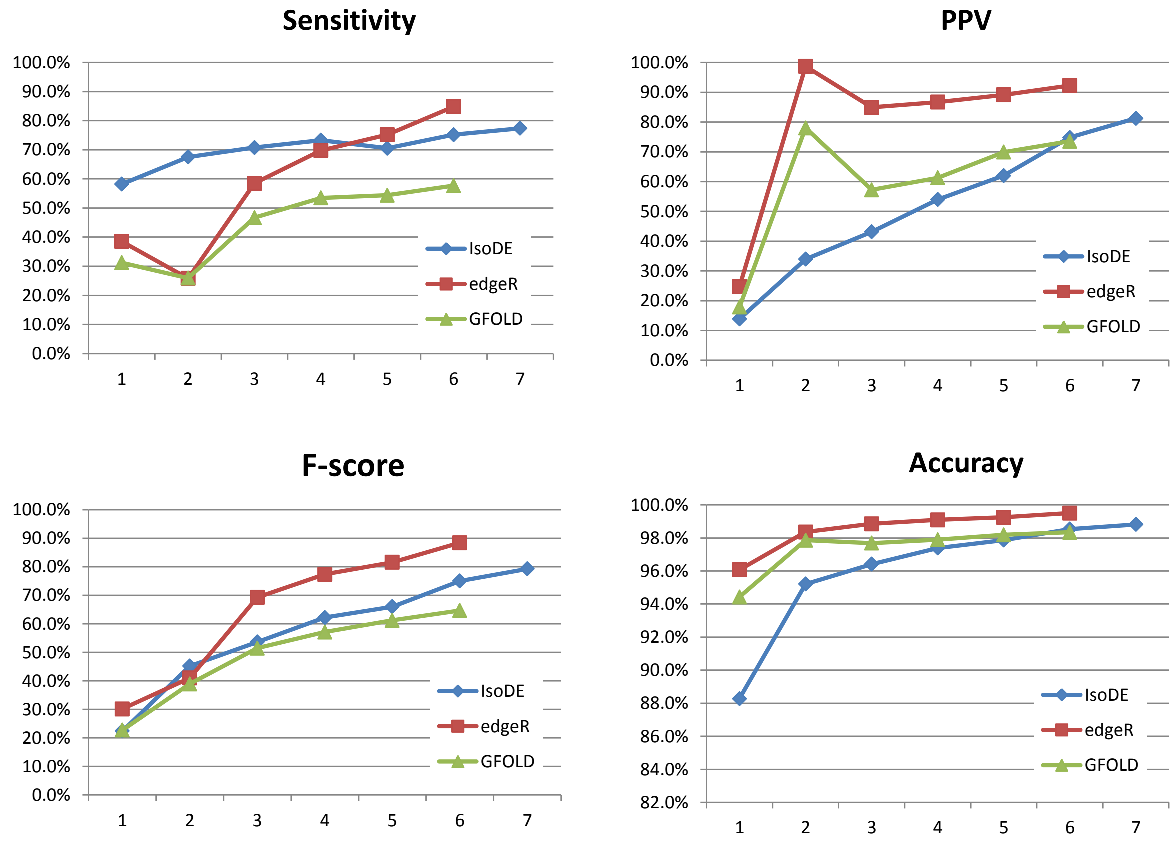

Figure S2: Sensitivity, PPV, F-Score, and accuracy of IsoDE-All (with 20 bootstrap runs per condition), edgeR, and GFOLD on the Illumina MCF-7 data with varying number of replicates and minimum fold change 2.

Data set	Sample	Reads Length	Mapped Reads*	Uniquely Mapped Reads*	Notes
SRX365211	MCF-7 Control	49	24.22	18.57	MCF-7 Control Replicate 1
SRX365212	MCF-7 Control	49	41.85	31.98	MCF-7 Control Replicate 2
SRX365213	MCF-7 Control	49	32.49	24.63	MCF-7 Control Replicate 3
SRX365214	MCF-7 Control	49	32.86	25.13	MCF-7 Control Replicate 4
SRX365215	MCF-7 Control	49	33.05	25.18	MCF-7 Control Replicate 5
SRX365216	MCF-7 Control	49	40.62	30.90	MCF-7 Control Replicate 6
SRX365217	MCF-7 Control	49	64.34	49.05	MCF-7 Control Replicate 7
SRX365204	MCF-7 E1	49	28.58	21.58	MCF-7 E2 Replicate 1
SRX365205	MCF-7 E2	49	23.71	18.26	MCF-7 E2 Replicate 2
SRX365206	MCF-7 E3	49	26.75	20.92	MCF-7 E2 Replicate 3
SRX365207	MCF-7 E4	49	24.95	18.64	MCF-7 E2 Replicate 4
SRX365208	MCF-7 E5	49	28.35	21.52	MCF-7 E2 Replicate 5
SRX365209	MCF-7 E6	49	27.08	20.80	MCF-7 E2 Replicate 6
SRX365210	MCF-7 E7	49	30.66	23.38	MCF-7 E2 Replicate 7
SRX003926	MAQC HBRR	36	6.80	4.8	MAQC Illumina dataset
SRX003927	MAQC UHRR	36	5.80	3.9	MAQC Illumina dataset
SRX002934	MAQC UHRR	253.4	0.52		MAQC First 454 dataset
SRX002935	MAQC HBRR	251.1	0.50		MAQC First 454 dataset
SRX002932	MAQC UHRR	253.6	0.54		MAQC Second 454 dataset
SRX002933	MAQC HBRR	252.0	0.78		MAQC Second 454 dataset
DID-143-282	MAQC HBRR	94.2	1.22		MAQC ION Torrent
LUC-140-265	MAQC HBRR	97.7	1.14		MAQC ION Torrent
GOG-139-281	MAQC HBRR	93.3	1.21		MAQC ION Torrent
LUC-141-267	MAQC HBRR	95.1	1.04		MAQC ION Torrent
POZ-124-266	MAQC HBRR	95.5	1.07		MAQC ION Torrent
DID-143-283	MAQC UHRR	99.8	1.37		MAQC ION Torrent
GOG-140-284	MAQC UHRR	101.1	1.45		MAQC ION Torrent
POZ-125-268	MAQC UHRR	96.8	1.10		MAQC ION Torrent
POZ-126-269	MAQC UHRR	102.1	1.29		MAQC ION Torrent
POZ-127-270	MAQC UHRR	99.8	1.62		MAQC ION Torrent

Table S1: Datasets used in the experimental analysis and their mapping statistics. Number of Uniquely mapped reads is specified for the datasets analyzed by edgeR. *In million reads

Fold Change	Method	Accuracy %	Sensitivity %	PPV %	F-Score %
1	FisherTotal	34.01%	30.49%	95.63%	46.24%
	FisherHousekeeping	24.53%	20.12%	**97.74%**	33.38%
	GFOLD	55.62%	54.18%	92.11%	68.23%
	IsoDE-Match	**78.39%**	**83.13%**	79.56%	**81.30%**
	IsoDE-All	78.10%	82.66%	79.23%	80.91%
1.5	FisherTotal	48.18%	35.37%	89.81%	50.75%
	FisherHousekeeping	42.48%	24.86%	**97.74%**	39.63%
	GFOLD	62.19%	58.13%	85.39%	69.17%
	IsoDE-Match	**71.09%**	**79.35%**	79.20%	**79.27%**
	IsoDE-All	70.36%	78.59%	79.04%	78.81%
2	FisherTotal	57.96%	39.53%	85.43%	54.05%
	FisherHousekeeping	55.33%	29.30%	**97.67%**	45.08%
	GFOLD	69.05%	61.16%	83.49%	70.60%
	IsoDE-Match	**74.60%**	**80.47%**	78.10%	**79.27%**
	IsoDE-All	71.97%	79.30%	75.61%	**77.41%**

Table S2: Accuracy, sensitivity, PPV and F-Score in % for the Second 454 dataset and fold change threshold f of 1, 1.5, and 2. The number of bootstrap samples is $M = 200$ for IsoDE-Match and $M = 20$ for IsoDE-All, and bootstrap support was determined using the binomial model with significance level $\alpha = 0.05$.

Fold Change	Method	Accuracy %	Sensitivity %	PPV %	F-Score %
1	FishersTotal	49.05%	46.44%	97.09%	62.83%
	FishersHousekeeping	40.88%	37.62%	**98.38%**	54.42%
	FisherERCC	52.55%	51.70%	88.83%	65.36%
	GFOLD	59.27%	59.29%	91.41%	71.92%
	IsoDE-All	**79.12%**	**83.75%**	79.91%	**81.78%**
1.5	FisherTotal	60.29%	53.35%	90.29%	67.07%
	FisherHousekeeping	57.08%	45.31%	**96.34%**	61.64%
	FisherERCC	57.96%	56.02%	82.07%	66.59%
	GFOLD	67.01%	65.58%	87.72%	75.05%
	IsoDE-All	**72.55%**	**80.50%**	80.34%	**80.42%**
2	FisherTotal	69.05%	59.53%	86.78%	70.62%
	FisherHousekeeping	68.90%	53.49%	**94.26%**	68.25%
	FisherERCC	66.42%	60.23%	80.93%	69.07%
	GFOLD	72.85%	66.51%	86.93%	75.36%
	IsoDE-All	**76.64%**	**81.40%**	81.02%	**81.21%**

Table S3: Accuracy, sensitivity, PPV and F-Score in % for ION Torrent pair HBRR: LUC-140_265 and UHRR: POZ-126_269, with fold change threshold f of 1, 1.5, and 2. The number of bootstrap samples for IsoDE-All is $M = 20$, and bootstrap support was determined using the binomial model with significance level $\alpha = 0.05$.

Fold Change	Method	Accuracy %	Sensitivity %	PPV %	F-Score %
1	FishersTotal	51.09%	48.76%	96.04%	64.68%
	FishersHousekeeping	46.42%	43.65%	**97.58%**	60.32%
	FisherERCC	55.04%	54.33%	88.86%	67.43%
	GFOLD	60.44%	60.84%	83.09%	70.24%
	IsoDE-All	**79.56%**	**84.37%**	80.50%	**82.39%**
1.5	FisherTotal	62.34%	56.02%	90.43%	69.19%
	FisherHousekeeping	61.61%	52.20%	**94.46%**	67.24%
	FisherERCC	60.44%	57.17%	85.92%	68.66%
	GFOLD	64.09%	62.91%	82.87%	71.52%
	IsoDE-All	**74.60%**	**80.69%**	82.10%	**81.39%**
2	FisherTotal	69.05%	60.47%	85.81%	70.94%
	FisherHousekeeping	70.36%	58.84%	**90.03%**	71.17%
	FisherERCC	67.30%	60.00%	82.96%	69.64%
	GFOLD	72.55%	65.12%	86.96%	74.45%
	IsoDE-All	**76.50%**	**79.77%**	81.28%	**80.52%**

Table S4: Accuracy, sensitivity, PPV and F-Score in % for ION Torrent pair HBRR: GOG-139_281 and UHRR: POZ-127_270, with fold change threshold f of 1, 1.5, and 2. The number of bootstrap samples for IsoDE-All is $M = 20$, and bootstrap support was determined using the binomial model with significance level $\alpha = 0.05$.

# Replicates	Rep1	Rep2	Rep3	Rep4	Rep5	Rep6	Rep7	Bootstraps Per Condition
1	20							20
2	10	10						20
3	7	7	6					20
4	5	5	5	5				20
5	4	4	4	4	4			20
6	4	4	3	3	3	3		20
7	3	3	3	3	3	3	2	20

Table S5: IsoDE setup for experiments with replicates. IsoDE experiments on the MCF-7 dataset was performed as follows. First we generated, for each of the 7 replicates of each condition 20, 10, 6, 5, 4, 3, respectively 2 bootstrap samples. We then used subsets of these bootstrap samples as input for IsoDE to perform DE analysis with varying number of replicates and a fixed total number $M = 20$ of bootstrap samples per condition. In experiment 1 we used 20 bootstrap samples from first replicate of each condition, in experiment 2 we used 10 bootstrap samples for each of the first 2 replicates of each condition, and so on.
